# Potential inhibitory effect of indolizine derivatives on the two enzymes: nicotinamide phosphoribosyltransferase and beta lactamase, a molecular dynamics study

**DOI:** 10.1007/s00894-017-3363-3

**Published:** 2017-06-20

**Authors:** Beata Szefler, Przemysław Czeleń

**Affiliations:** Department of Physical Chemistry, Faculty of Pharmacy, Collegium Medicum, Nicolaus Copernicus University, Kurpińskiego 5, 85-096 Bydgoszcz, Poland

**Keywords:** Beta lactamase, Nicotinamide phosphoribosyltransferase, Indolizine, Docking, Molecular dynamics, Affinity energy

## Abstract

Nicotinamide phosphoribosyl-transferases (NAMPT) are enzymes that play a role in targeting cancer metabolism, while beta lactamases are involved in bacterial resistance to beta-lactam antibiotics. Many protein inhibitors exhibit such property which is often correlated with their cellular potency. In order to understand such a phenomenon, the present article conducts an analysis of the dynamic behavior of complexes formed by the inhibitors, that is indolizine derivatives, with the studied enzymes. Both docking and molecular dynamics led to identification of their interactions and showed the mechanism of inhibition of the two studied enzymes. The differences in the behavior of ligand at the active sites of beta lactamases and nicotinamide phosphoribosyl-transferases are indicated by structural and enthalpy values.

**Electronic supplementary material:**

The online version of this article (doi:10.1007/s00894-017-3363-3) contains supplementary material, which is available to authorized users.

## Introduction

**Fig. 1:**
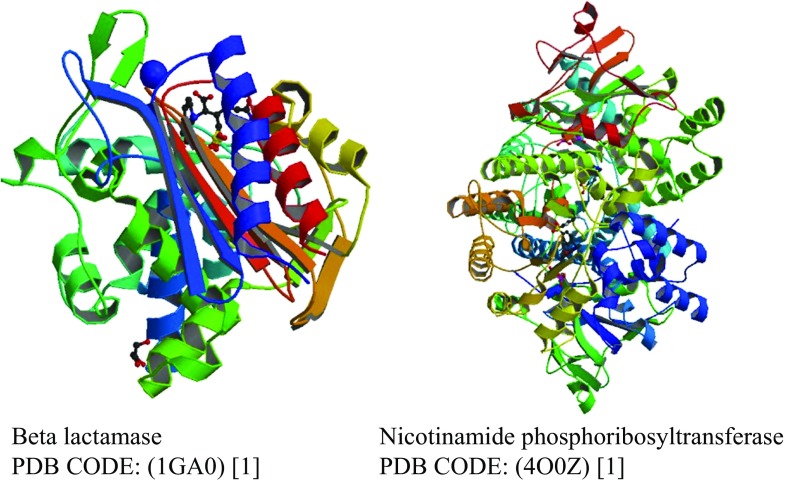
The proteins studied in this paper: beta lactamase and nicotinamide phosphoribosyltransferase (RCSB PDB code: 1GA0; 4O0Z)

Nicotinamide phosphoribosyl-transferases (NAMPT) are enzymes that play a role in targeting cancer metabolism (Fig. 1, right) [1]. Many NAMPT inhibitors exhibit such property which is often correlated with their cellular potency, namely they undergo NAMPT-catalyzed phosphoribosylation (pRib). In order to understand this phenomenon, the present article conducts an analysis of the dynamic behavior of complexes formed by the proposed inhibitors (indolizine derivatives) with Nicotinamide phosphoribosyl-transferase. The possible mechanism of ligand-protein interaction is described and discussed in the course of the study. Also, studying the ligand–beta lactamase affinity seems to be interesting and was therefore conducted. Beta lactamases have the ability of breaking the beta-lactam ring, disabling the action of penicillin-like antibiotics and are involved in bacterial resistance to beta-lactam antibiotics (Fig. 1, left) [2]. The description of the energetic and geometric features of the studied ligand binding to the active site of the enzyme may be useful for understanding the mechanism of the ligand-enzyme interaction.

Indolizine derivatives were proposed as the inhibitors of both proteins presented above. These derivatives are heteroaromatic compounds of pharmacological importance with two condensed (5- and 6-memebered) rings bridged by a nitrogen atom. They can inhibit enzyme activity and act as calcium entry blockers in cardiovascular activity [3]. Also, their biological activities as antimicrobial, antioxidant, anti-inflammatory, tuberculostatic, and anticonvulsant agents were discovered [3]. Synthesis mechanisms of indolizines involve 1,3-dipolar cycloadditions, cyclization reactions, etc. [4]. Indolizines are an important group of compounds also because of their behavior as histamine H3 receptor antagonists, 5-HT3 receptor antagonists, and antitumorals [5]. In nature they have been isolated from animals, insects, plants, marine organisms, and microbes [6].

The present study includes three steps in the methodological procedure: docking, molecular dynamics simulation and calculating Gibbs free energy [7, 8].

The docking procedure was applied to a set of 20 indolizines, downloaded from the PubChem database [9]. Beta lactamase (PDB code: 1GA0, Fig. 1, left) and nicotinamide phosphoribosyltransferases (PDB code: 4O0Z, Fig. 1, right) downloaded from RCSB protein data bank [1] were considered for potential binding affinity with selected indolizine derivatives.

After applying the docking procedure only one of 20 tested indolizine molecules was selected for further studies. Application of docking and molecular dynamics allows studing the enzyme-ligand interactions in a large number of their conformations, in their natural environment [10–13]. Carrying out molecular dynamics allows for the identification and the specification of interactions responsible for the stabilization of complexes of the chosen ligand (i.e., indolizine derivative) with beta lactamase and nicotinamide phosphoribosyltransferases. The behavior of the ligand at the active sites of the investigated enzymes is indicated by structural and energetic (enthalpy values) data. The previous study, based on docking procedure and molecular dynamics simulations, revealed the mechanism of inhibition of the two above enzymes, where the proposed inhibitor was one of the indolizine derivatives.

## Materials and methods

### Docking procedure

The docking procedure was applied with the use of AutoDockVina software by utilizing united-atom scoring function [14]. The protein molecules in the form of “protein.pdb” files were loaded from Brookhaven Protein Database PDB [1]. Before the docking procedure hydrogen bonds were removed [15]. The investigated ligands were loaded from PubChem Database [9]. Their torsions along the rotatable bonds were assigned, and then the files were saved as “ligand.pdbqt”. Before the docking procedure, all water molecules were also removed from crystal structures of the enzymes. Both ligands and the proteins held only polar hydrogen atoms. All preparation steps were realized using Auto Dock Tools package. Docking parameter files were completed by using the Lamarckian genetic algorithm [16] where the grid menu was toggled [17]. The correctness of the applied algorithm was verified by crystal structures. A docking algorithm was applied in the case of all considered proteins. After loading “protein.pdbqt”, the map files were selected directly with setting up the grid points for the search of ligand-protein interactions, separately for each protein. Before the docking procedure a grid box with dimensions 24x24x24 and the exhaustiveness value of 15 was used. After the docking procedure the analysis of nine ligand–protein conformations was conducted.

### Molecular dynamics method

Based on the energetic and structural analysis of docked enzyme–ligand complexes [15, 17] there was applied a molecular dynamics (MD) procedure [18]. The docking procedure provided enzyme-ligand complexes with the best energy of interactions and at the same time with the highest number of bonds. In this aspect bond strength was analyzed in structural terms. These complexes served as a starting point for molecular dynamics. The Amber force field parameter ff14SB was used for parametrization of studied enzymes, and Gaff parameter in the case of ligands [19]. The atomic charges were calculated according to the Merz-Kollmann scheme, via the RESP procedure [20] at HF/6-31G* level of theory. Each system was neutralized with the use of ions (1GA0–2 chloride anions ∼6 mM with Joung and Cheatham [21, 22] parameters set, 4O0Z - 6 sodium cations ∼8 mM) and immersed in a periodic TIP3P water box. Both systems were minimized and this process was realized in two stages with the use of steepest descent and conjugate gradient methods. After minimization each system was heated up to 300 K by 100 ps of initial MD simulation. To control the temperature the Langevin thermostat was used [23]. The periodic boundary conditions and SHAKE algorithm [24] were applied to 70 ns of molecular dynamics simulation. The first 10 ns of the simulation time were considered as the equilibration interval while the next 60 ns of trajectory were used for the analysis of ligand-enzyme interactions. Structural analysis was performed by the VMD package [25]. The MM/PBSA and MM/GBSA methods were used for estimating the values of the binding free energy. The polar desolvation free energy was estimated by the GB model, developed by Onufriev et al. in the case of MM/GBSA calculations [26] and by the PB solver implemented in the PBSA module in the case of MM/PBSA calculations [27]. Atoms optimized with radii optimization by Tan and Luo were used during calculations realized in the TIP3P explicit solvent [28]. Because of expensive computational cost and no significant improvement of results, the entropic contribution to Gibbs free energy was omitted in many cases [29–32]. In MD simulations, the AMBER 14 package was used [18].

## Results and discussion

### Docking results

**Fig. 2:**
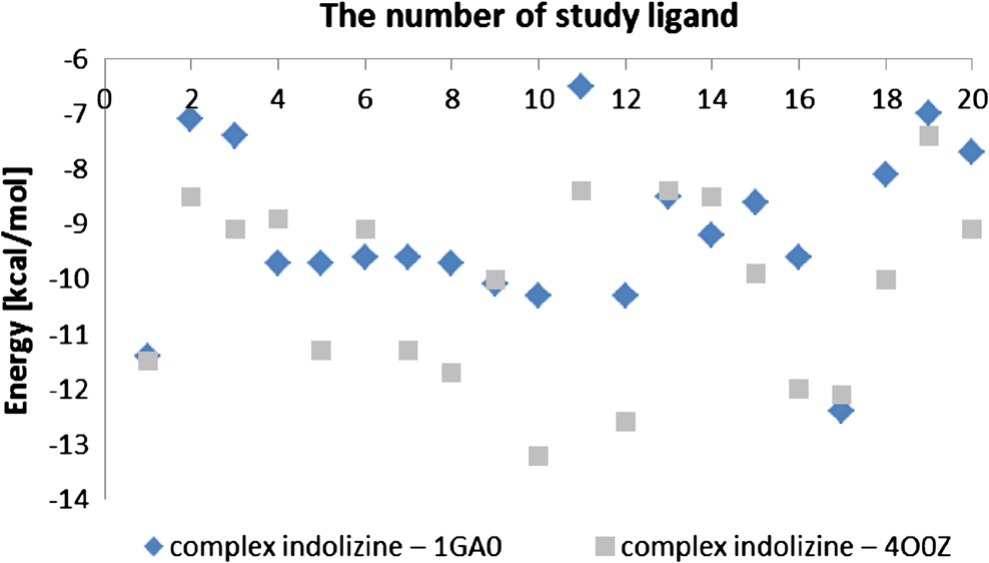
Energy (in kcal mol^−1^) of the best affinity ligand (indolizine deratives) – enzyme (beta lactamase and nicotinamide phosphoribosyltransferase PDB code: 1GA0 and 4O0Z)

**Fig. 3:**
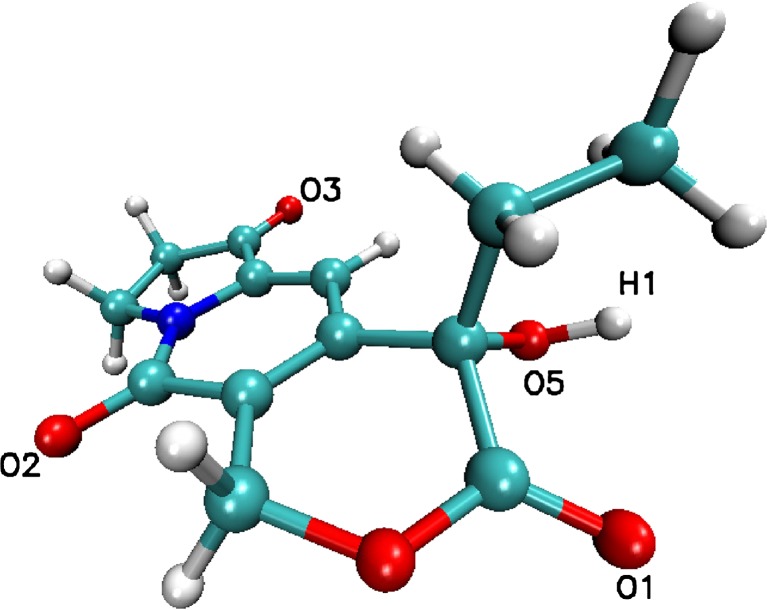
Structure of the studied ligand with PDB code: 359,849, marked atoms are involved in creation of hydrogen bonds with amino acids from active sites of beta lactamase (PDB code: 1GA0) and nicotinamide phosphoribosyltransferase (PDB code: 4O0Z)

The docking procedure was applied to a set of 20 indolizines, downloaded from the PubChem database [9]. Beta lactamase (1GA0, Fig. 1 left, Fig. 2, Table 1) and nicotinamide phosphoribosyltransferases (4O0Z, Fig. 1 right, Fig. 2, Table 1) were considered for potential binding affinity with selected indolizine derivatives. The docking analysis, which was conducted on 20 molecules (Fig. 2, Table 1) provided data of affinity energy in the range from (−12.4) to (−6.5) kcal mol^−1^ for beta lactamase (1GA0) and from (−13.2) to (−7.4) for nicotinamide phosphoribosyltransferase (4O0Z). The considered ligand-enzyme complexes recreate with good accuracy the position of the co-crystallized ligand [7, 8]. The best binding energies, that is (−12.4 kcal mol^−1^) in the case of beta lactamase and (−13.2 kcal mol^−1^) in the case of nicotinamide phosphoribosyltransferase, were reached for two indolizine derivatives characterized by PDB code: 4,123,812 and PDB code: 359,849, respectively [9]. The hydrogen bonds (HB) play an important role in stabilization of the enzyme–ligand complex. The hydrogen bonds Y ... H…X are classified as the function of the distance d(Y,H) between the acceptor Y and hydrogen atom H, where X is a donor [33]. Detailed qualitative and quantitative analysis of hydrogen bonds formed between the ligand and the protein after docking procedure led to the selection of only one of the tested indolizine molecules for further studies. The indolizine with PDB code: 359,849 was used as the input for the next MD study (Fig. 3).

**Table 1:** Lamarckian genetic algorithm docked state – the best binding energy (kcal mol^−1^) of ligand indolizine derivatives binding to the active sites of type A of beta lactamase PDB code: 1GA0 and nicotinamide phosphoribosyltransferase PDB code: 4O0Z

Number of study ligand	Ligand PDB code	ΔE_1GA0_	ΔE_4O0Z_
1	42,783	**−11.4**	−11.5
2	47,673	−7.1	−8.5
3	47,680	−7.4	−9.1
4	202,885	−9.7	−8.9
5	223,313	−9.7	−11.3
6	243,979	−9.6	−9.1
7	346,940	−9.6	−11.3
8	346,948	−9.7	−11.7
9	356,969	−10.1	−10
10	359,849	**−10.3**	***−13.2***
11	583,044	−6.5	−8.4
12	625,271	−10.3	−12.6
13	853,043	−8.5	−8.4
14	2,060,443	−9.2	−8.5
15	3,056,676	−8.6	−9.9
16	3,739,820	−9.6	−12
17	4,123,812	***−12.4***	**−12.1**
18	5,272,726	−8.1	−10
19	22,565,707	−7	−7.4
20	45,099,172	−7.7	−9.1

**Fig. 4:**
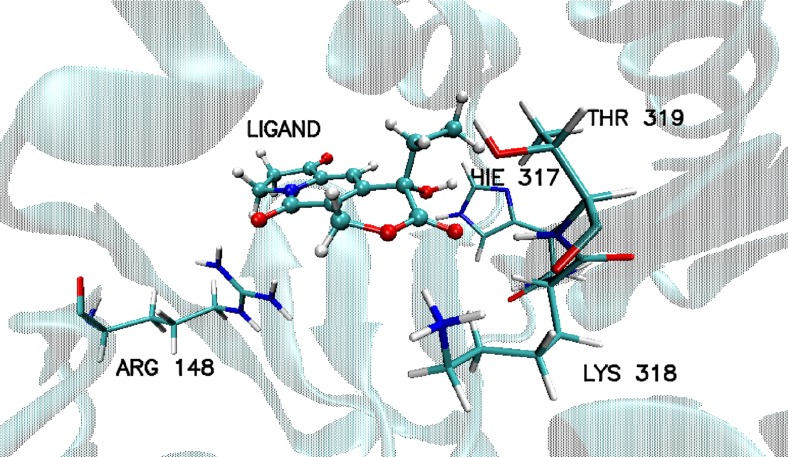
Basic interactions observed in the complex of ligand PDB code: 359,849 and beta lactamase PDB code: 1GA0; after docking procedure

**Fig. 5:**
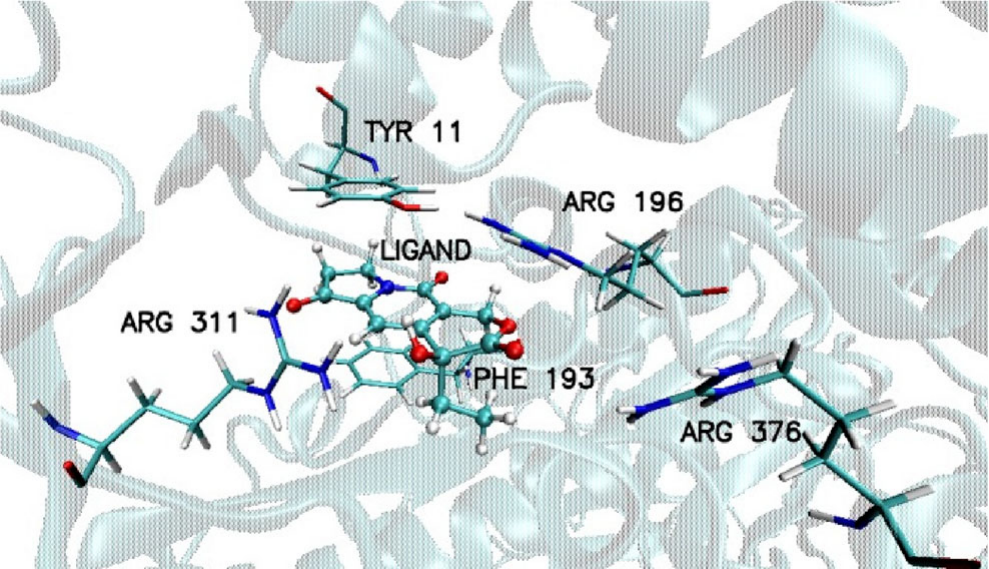
Basic interactions observed in the complex of ligand PDB code: 359,849 and nicotinamide phosphoribosyltransferase PDB code: 4O0Z; after docking procedure

After docking procedure the amino acids of 1GA0 involved in HB formation at the active site (Fig. 4) were identified as: LYS317, THR319, HIE317, ARG148, where in all cases the acceptor is derived from amino acid and the donor from ligand, (LYS317(H)…(O4)ligand, THR319(H)…(O1)ligand, HIE317(H)…(O5)ligand), ARG148(H)…(O2)ligand, Fig. 4). The same type of HB formation (amino acid(H)…(O)ligand) was observed in the case of nicotinamide phosphoribosyltransferase (4O0Z), TYR11(H)…(O3)ligand, ARG311(H)…(O3 and O5) ligand, ARG376(H)…(O1)ligand, ARG196(H)…(O1)ligand. Also, after docking evidence was found of stacking interactions between aromatic rings PHE193, TYR11 of 4O0Z, and aromatic rings of ligand, which together with hydrogen bonds play a significant role in stabilization of ligand in the pocket of protein (Fig. 5). Corresponding to the stacking of aromatic rings of TYR11 and ligand an additional effect appeared, coming from the hydroxyl group of the studied ligand, namely TYR11(H)…(O3)ligand.

### Molecular dynamics results

**Fig. 6:**
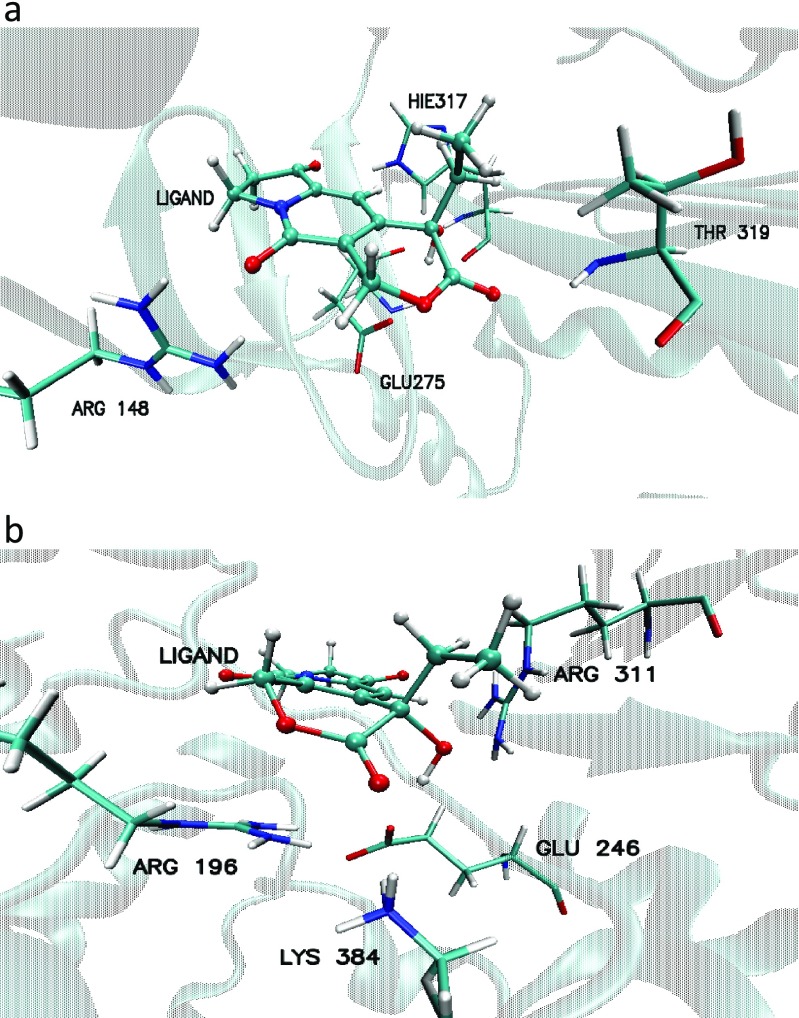
Basic interactions observed in the complex of ligand PDB code: 359,849 and the active site/pocket of proteins beta lactamase PDB code: 1GA0(a); nicotinamide phosphoribosyltransferase PDB code: 4O0Z (b) after molecular dynamics

**Fig. 7:**
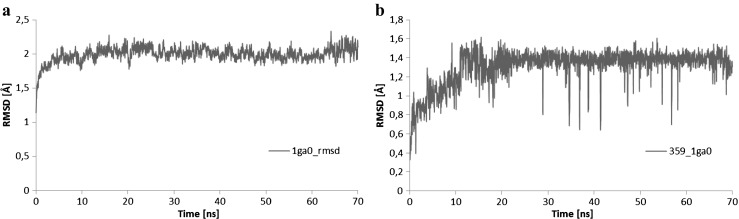
Distribution of RMSD in the ligand PDB code: 359,849 (a) and in the beta lactamase PDB code:1GA0 protein (b) values characterizing the interaction of ligand at the active site of 1GA0 enzyme

**Fig. 8:**
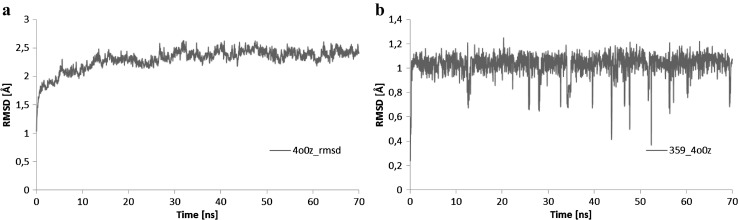
Distribution of RMSD in ligand PDB code: 359,849 (a) and in the nicotinamide phosphoribosyltransferase PDB code:4O0Z protein (b) values characterizing the interaction of ligand at the active site of 4O0Z enzyme

Application of docking and molecular dynamics allows the study of enzyme-ligand interactions in a large number of their conformations, in their natural environment [11]. Here, the interactions of the ligand with two enzymes, beta lactamase nicotinamide and phosphoribosyltransferase (PDB code: 1GA0 and 4O0Z), in their active sites in water solution were described. The 60 ns of trajectory of molecular dynamics were used for structural analysis. The studied ligand, due to the number and quality of hydrogen bonds (HBs) which formed with amino acids, is stable in the pockets of enzymes throughout all molecular dynamics simulations. The interactions formed between the amino acids of enzymes beta lactamase nicotinamide and phosphoribosyltransferase (PDB code: 1GA0 and 4O0Z) and ligand PDB code: 359,849 are presented in Fig. 6. The mean root-mean-square deviation (RMSD) values were used for the identification of the stability of studied enzyme-ligand complexes during the MD trajectories. The time evolutions of RMSDs for the ligand molecule and the proteins are shown in Figs. 7 and 8, as well as Table 2. The evolution of ligand in complex with 1GAO, as given by the RMSD values, seems to be stabilized after 20 ns of MD (Fig. 7, Table 2). Similarly, in the case of complex with 4O0z the equilibrium stage of ligand was reached during all molecular dynamics simulation (Fig. 8, Table 2). RMSD of ligand in the 1GA0–ligand complex increased from (1.134 ± 0.241 Å) to (1.307 ± 0.186 Å) in terms of average values, after stabilization (Table 2). RMSD of ligand in the 4O0z–ligand complex reached the value of 1.030 ± 0.095 Å. In both cases the standard deviation shows a rather low value (Table 2) which indicates that the mobility of ligand in the pockets of studied enzymes is quite small (Figs. 7, 8, and Table 2).

**Table 2:** Average RMSDs for the ligand and for the amino acids comprised in the active site, across the full MD simulation

	Ligand in the active site of complex with 1GA0	Ligand in the active site of complex with 4O0Z	The active site of 1GA0	The active site of 4O0Z
RMSD (Å)	1.307	1.030	1.993	2.303
SD	0.186	0.095	0.103	0.185

SD standard deviation

**Fig. 9:**
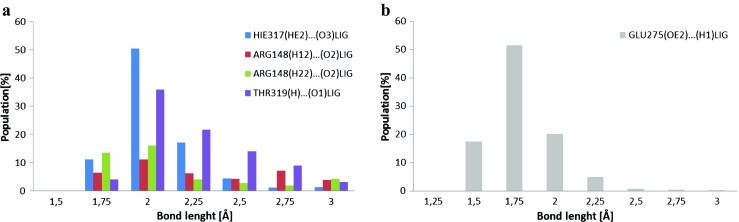
Distribution, obtained during the MD simulation, of the length of hydrogen bonds created by the interaction of ligand PDB code: 359,849 with selected amino acids from the active site of beta lactamase PDB code: 1GA0

According to the criterion “strong, average/medium and poor/low HB” [33], the proteins beta lactamase (1GA0) and nicotinamide phosphoribosyltransferase (4O0Z) create low, medium, and strong HBs with the studied ligands after molecular dynamics (MD). The amino acids of 1GA0 involved in HB formation at the active site (Fig. 9, Table 3) include: GLU275, HIE317, THR319, ARG148, LYS318, SER 64, and SER292. They form two kinds of HBs with the ligand where acceptor can be derived from amino acid and donor from ligand and vice versa, that is (amino acid(H)…(O)ligand, Fig. 9a, Table 3) and (amino acid(O)…(H)ligand, Fig. 9b, Table 3) respectively, with different strength: GLU275(O2)…(H1)LIG, HIE317(HE2)…(O3)LIG, ARG148(H12)…(O2)LIG, ARG148(H22)…(O2)LIG, THR319(H)…(O1)LIG (Fig. 9). As well as in the case of ARG148, LYS318, SER 64, and SER292, they create hydrogen bonds during very short times of molecular simulations and that is why these interactions are irrelevant. Population of hydrogen bonds (providing info about their strength) is given in % of the MD simulation time, e.g., GLU275(O2)…(H1)LIG, 96%; HIE317(HE2)…(O3)LIG, 86%, ARG148(H12)…(O2)LIG,39.35%, ARG148(H22)…(O2)LIG, 42.9%, THR319(H)…(O1)LIG, 88.12% (Table 3). As can be seen, the creation of hydrogen bonds with three amino acids GLU275, THR319, and HIE317 is responsible for stabilization of ligand in the pocket of protein 1GA0 after molecular dynamics (Table 3). HIE317, ARG148, and THR319 create low, medium and strong HBs with the studied ligand (Table 3) and GLU275 forms strong and medium, respectively (Table 3). During over 50% of simulation of MD the hydrogen atom (HE2) of HIE317 interacts with oxygen atom (O3) of ligand with bond length 2 Å, during about 17% of MD with 2.25 Å and during 11% with 1.75 Å, respectively. THR319 forms HBs with ligand with mostly medium strength (Fig. 9, Table 3). ARG148 forms bonds with the ligand during all of the dynamics time however without a dominating one, but oxygen atom (O2) of GLU275 forms strong HBs with hydrogen atom (H1) of ligand during 50% of MD with 1.75 Å bond length, during 17.5% with 1.5 Å bond length, and during about 20% with 2 Å (Fig. 9, Table 3).

**Table 3:** Distributions of the most frequently created hydrogen bonds between ligand PDB code: 359,849 and selected amino acids from beta lactamase (PDB code: 1GA0) active sites. Hydrogen bonds in the table represent middle values of intervals with width of 0.25 Å

Hydrogen bond	Length of hydrogen bond [Å]	Population %
Beta lactamase (PDB code: 1GA0)		
GLU275(**OE2**)…(**H1**)LIG	1.5 1.75 2 2.25 2.5	17.5 51.5 20.3 5.1 0.9
HIE317(**HE2**)…(**O3**)LIG	1.75 2 2.25 2.5 2.75 3	11.2 50.4 17.2 4.5 1.25 1.45
THR319(**H**)…(**O1**)LIG	1.5	0.05
	1.75	4.14
	2	35.85
	2.25	21.75
	2.5	14.15
	2.75	9
	3	3.18
ARG148(**H12**)…(**O2**)LIG	1.75 2 2.25 2.5 2.75 3	6.4 11.2 6.25 4.25 7.25 4
ARG148(**H22**)…(**O2**)LIG	1.5	0.2
	1.75	13.5
	2	16.1
	2.25	4.15
	2.5	2.75
	2.75	1.95
	3	4.25

**Fig. 10:**
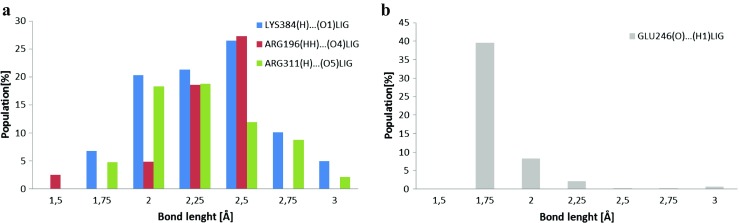
Distribution, obtained during the MD simulation, of the length of hydrogen bonds, created by ligand PDB code: 359,849 with selected amino acids from the active site of nicotinamide phosphoribosyltransferase PDB code: 4O0Z

The study confirms literature data and demonstrates the application of docking procedure compliance for 1GA0. The following amino acids of proteins interact with the inhibitor: SER64 and THR319 [34]. There were found the contributions of the same amino acids in forming hydrogen bonds after docking and molecular dynamics, that is HIE317, THR319, ARG148, LYS318. Interestingly, the analysis of trajectories of MD showed a new very important HB which participates in stabilization of ligand in the pocket of enzyme, that is GLU275(O2)…(H1)LIG with 99.6% of population of MD, which was not seen after docking procedure. In the case of HIE317, amino acid creates HB not with the oxygen atom (O5) of hydroxyl group of ligand as it had been after docking procedure, but with the O3 oxygen atom of ligand. In the case of enzyme 4O0Z, the four amino acids: GLU246, ARG196, ARG311, LYS384, are involved in the formation of HBs with the ligand (Figs. 6b and 10), and again, similarly as with 1GA0, they create two kinds of HBs with the ligand where acceptor can come from amino acid and donor from ligand and vice versa (Fig. 10). At about 90% of analyzed trajectories of molecular dynamics hydrogen atom (H) of LYS384 forms hydrogen bonds with oxygen atom (O1) of ligand over the range of impact: during over 5% of simulation with bond length 1.75 Å and 3 Å independently, over 20% with 2 Å, 2.25 Å, and 2.5 Å and during about 10% of trajectory with 2.75 Å bond length (Fig. 10a, Table 4). A similar distribution of percentages for the length of created hydrogen bonds with slightly reduced values is observed for ARG311 (Fig. 10a, Table 4). This hydrogen bond symbolized ARG196(H)…(O4)LIG manifested during almost 45% of the cumulative time of MD simulations with a medium strength (2.75 Å and 3 Å, Fig. 10a and Table 4). On the contrary, in (GLU246(O)…(H1)LIG) there were detected strong HBs during 50% of the cumulative time of simulation (1.5 Å and 1.75 Å, Fig. 10b and Table 4). Due to the fact that the tested ligand PDB code: 359,849 was in a deeper position in the pocket of enzyme, during the study there were found other amino acids which formed hydrogen interactions with the ligand in the active site of 4O0Z, compared with literature data [35]. Even so, the analysis of molecular dynamics shows that two of five amino acids play the same role in stabilization of ligand in pocket of protein as it was after docking procedure, that is ARG196, ARG311. The active site of 4O0Z protein (Fig. 6b, Table 5) creates hydrophobic interactions with the aromatic rings of ligand, classified as stacking interactions. This type of interactions was found after docking procedure and during over 90% of molecular dynamics simulations. These interactions involve two aromatic rings derived from TYR11 and PHE193, and the aromatic ring of the ligand is seated between them (Fig. 10, Table 4). The distributions of hydrogen bond (HB) formed between the ligand PDB code: 359,849 and enzymes: the beta lactamase PDB code:1GA0 protein and nicotinamide phosphoribosyltransferase PDB code:4O0 protein are shown in Figs. 11 and 12 in the Supplementary material. In Table 6 the data of enthalpy calculation for the two ligand–enzyme complexes are given. These data confirm that the ligand–1GA0 complex is much more stable, compared with ligand–4O0Z complex. This observation is confirmed by the number and quality of ligand–enzyme interactions. In order to estimate enthalpy values of the analyzed systems two methods, namely MM/GBSA and MM/PBSA were used (Table 6). Obtained values for both methods are slightly different but the trends in their values are preserved (Table 6).

**Table 4:** Distributions of the most frequently created hydrogen bonds between ligand PDB code: 359,849 and selected amino acids from nicotinamide phosphoribosyltransferase (PDB code: 4O0Z) active sites. Hydrogen bonds in the table represent middle values of intervals with width of 0.25 Å

Hydrogen bond	Length of hydrogen bond [Å]	Population %
Nicotinamide phosphoribosyltransferase (PDB code: 4O0Z)		
LYS384**(H)…(O1)**LIG	1.75 2.00 2.25 2.50 2.75 3.00	6.75 20.32 21. 26.45 10.12 4.98
GLU246**(O)…(H1)**LIG	1.5 1.75 2 2.25 2.5 2.75 3	12.86 39.56 8.23 2.15 0.21 0.23 0.59
ARG196**(HH)…(O4)**LIG	2.50 2.75 3.00	4.87 18.56 27.28
ARG311**(H)…(O5)**LIG	1.75 2.00 2.25 2.50 2.75 3.00	2.49 11.38 13.28 11.88 10.58 5.69

**Table 5:** Distributions of stacking interactions between aromatic ring of ligand PDB code: 359,849 and aromatic rings of nicotinamide phosphoribosyltransferase (PDB code: 4O0Z) active sites. Distance between two aromatic rings in table represents middle values of intervals with width of 0.25 Å

Stacking interaction	Distance between two aromatic rings [Å]	Population %
Nicotinamide phosphoribosyltransferase (PDB code: 4O0Z)		
TYR11(**CG**)…(**C10**)LIG	3.25 3.5 3.75 4 4.25 4.5 4.75	3.60 17.53 26.01 29.1 15.74 6.45 1.17
PHE193(**CE**)…(**C6**)LIG	3 3.25 3.5 3.75 4 4.25	6.53 40.34 38.91 12.41 2.56 0.20

**Table 6:** Enthalpy values for the ligand–enzyme complex (PDB code: 1GA0 and 4O0Z)

	1GA0		4O0Z	
	ΔE	*SD*	ΔE	*SD*
E_VDWAALS_	−36.85	3.68	−37.15	2.76
E_EL_	−45.85	6.76	−39.23	7.63
E_GB_	58.84	5.21	59.14	4.35
E_SURF_	−4.65	0.29	−3.45	0.18
ΔH_GB_	**−28.51**	**4.65**	**−20.69**	**3.84**
E_VDWAALS_	−36.85	3.68	−37.15	2.76
E_EL_	−45.85	6.76	−39.23	7.63
E_PB_	63.75	5.39	66.98	6.4
E_CAVITY_	−2.65	0.13	−2.38	0.17
ΔH_PB_	**−21.6**	**4.82**	**−11.78**	**6.45**

E_VDWAALS_ = van der Waals contribution from MM. E_EL_ = electrostatic energy, E_PB_/E_GB_ = the electrostatic contribution to the solvation free energy calculated by PB or GB respectively. E_CAVITY_ = nonpolar contribution to the solvation free energy. ΔH = final estimated binding enthalpy

## Conclusions

In a previous work a mechanism was described of interaction of one of the indolizine derivatives with PDB code: 359,849 with two enzymes, beta lactamase and nicotinamide phosphoribosyltransferase after docking procedure and molecular dynamics simulation. The RMSD values confirm that the studied indolizine derivative was stable with both analyzed proteins. Even so, the analysis of the strength and the number of hydrogen bonds between the studied ligand and the active sites of enzymes shows a higher affinity of the ligand to 1GA0, compared with 4O0Z, that is confirmed by enthalpy values of the analyzed systems. Based on the energetic and structural data presented above, the highest affinity of the studied ligand is manifested toward the enzyme 1GA0. The values of affinity energy after docking study have opposite conclusions compared with the molecular dynamic, which is associated with the fact that during the molecular dynamics, in over 90% of simulations, there appeared new strong hydrogen bonds for ligand–1GA0. For both beta lactamase and nicotinamide phosphoribosyltransferase contributions were found of the same amino acids in forming hydrogen bonds after docking and molecular dynamics. Results obtained after molecular dynamics simulations confirm literature data for beta lactamase [34]. In the case of nicotinamide phosphoribosyltransferase, due to the deeper location of the ligand in the enzyme pocket, completely different interactions of amino acids with inhibitor in comparison with literature data were found [35].

## Electronic supplementary material

ESM 1 (DOCX 213 kb)
