# Supplementary material for: Potential inhibitory effect of indolizine derivatives on the two enzymes: nicotinamide phosphoribosyltransferase and beta lactamase, a molecular dynamics study
Source: J Mol Model. 2017 Jun 20;23(7):208. doi: 10.1007/s00894-017-3363-3 (PMC5487893; doi:10.1007/s00894-017-3363-3)
Supplement: Supplementary file 1 — (DOCX 213 kb) [file 894_2017_3363_MOESM1_ESM.docx]

**SUPPLEMENTARY MATERIALS**

| 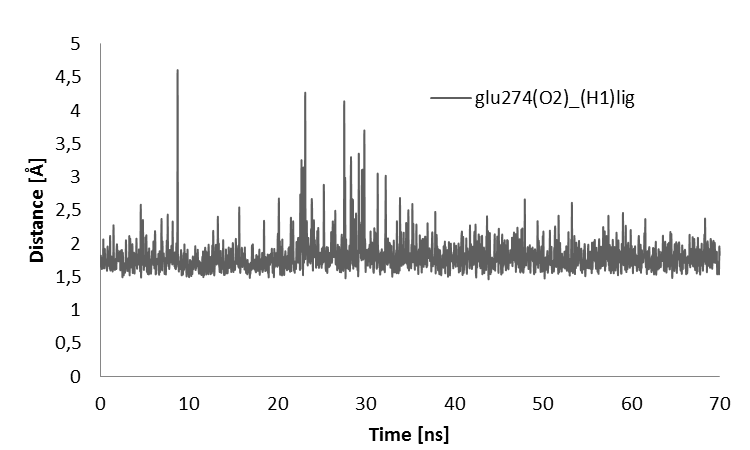 | **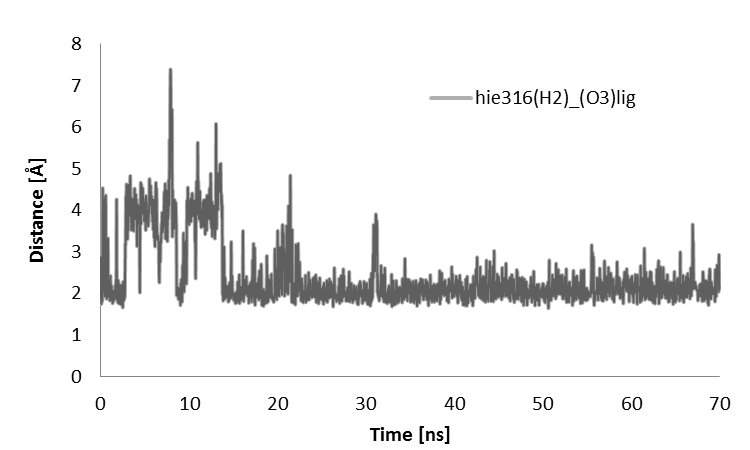** |
| --- | --- |
| **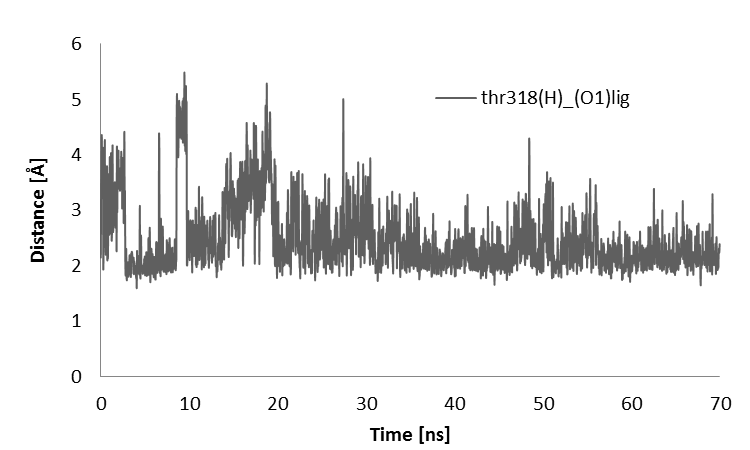** | |
| **Figure 11.** Distribution of Hydrogen Bond (HB) formed between the ligand PDB CODE: 359849 and the beta lactamase PDB CODE:1GA0 protein. | |
| **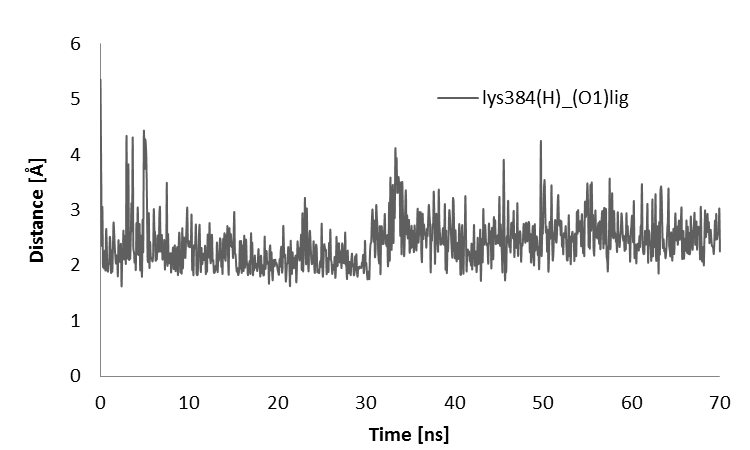** | **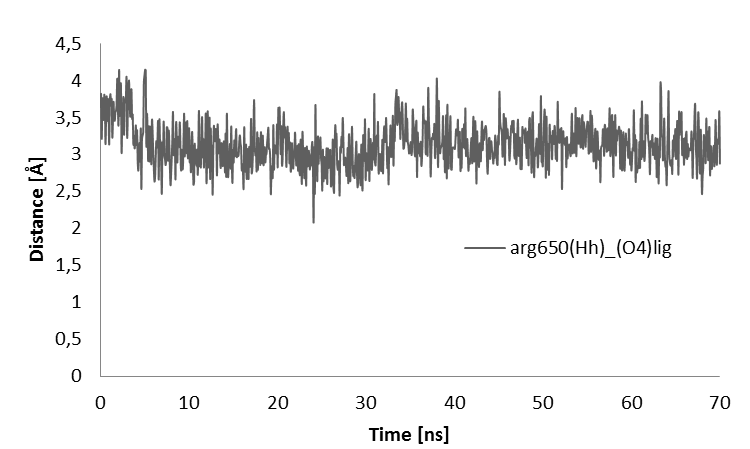** |
| **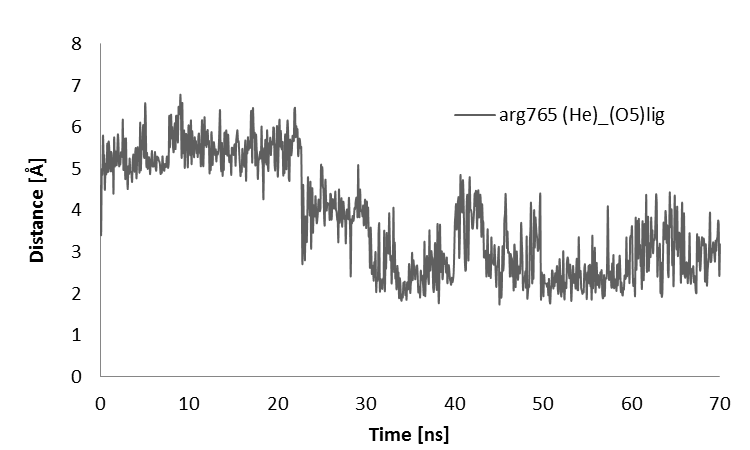** | |
|  | |

**Figure 12.** Distribution of Hydrogen Bond (HB) formed between the ligand PDB CODE: 359849 and nicotinamide phosphoribosyltransferase PDB CODE:4O0Z protein.
